# Neoadjuvant Cabozantinib in an Unresectable Locally Advanced Renal Cell Carcinoma Patient Leads to Downsizing of Tumor Enabling Surgical Resection: A Case Report

**DOI:** 10.3389/fonc.2020.622134

**Published:** 2021-02-01

**Authors:** Mehmet A. Bilen, James F. Jiang, Caroline S. Jansen, Jacqueline T. Brown, Lara R. Harik, Aarti Sekhar, Haydn Kissick, Shishir K. Maithel, Omer Kucuk, Bradley Carthon, Viraj A. Master

**Affiliations:** ^1^ Department of Hematology and Medical Oncology, Emory University, Atlanta, GA, United States; ^2^ Winship Cancer Institute of Emory University, Atlanta, GA, United States; ^3^ Department of Urology, Emory University, Atlanta, GA, United States; ^4^ Department of Pathology, Emory University, Atlanta, GA, United States; ^5^ Department of Radiology, Emory University, Atlanta, GA, United States; ^6^ Department of Surgery, Emory University, Atlanta, GA, United States

**Keywords:** cabozantinib, renal cell carcinoma, neoadjuvant therapy, radical nephrectomy, case report

## Abstract

**Introduction:**

Cabozantinib (XL-184) is a small molecule inhibitor of the tyrosine kinases c-Met, AXL, and VEGFR2 that has been shown to reduce tumor growth, metastasis, and angiogenesis. After the promising results from the METEOR and CABOSUN trials, cabozantinib was approved for use in the first- and second-line setting in patients with advanced RCC. Previously, targeted therapies have been used in the neoadjuvant setting for tumor size reduction and facilitating nephrectomies. The increased response rates with cabozantinib in metastatic renal cell carcinoma (mRCC), along with the other neoadjuvant TKI data, strongly support an expanded role for cabozantinib in the neoadjuvant setting.

**Case Description:**

We report on a 59-year-old gentleman presenting with an unresectable 21.7 cm left renal cell carcinoma (RCC) with extension to soft tissue and muscles of the thoracic cage, psoas muscle, posterior abdominal wall, tail of pancreas, splenic flexure of colon, and inferior margin of spleen. Presurgical, neoadjuvant systemic therapy with cabozantinib was initiated for 11 months in total. Initially after 2 months of cabozantinib, magnetic resonance imaging (MRI) revealed a significant reduction (44.2%) in tumor diameter from 21.7 to 12.1 cm with decreased extension into adjacent structures. After 11 months total of cabozantinib, the corresponding MRI showed grossly stable size of the tumor and significant resolution of invasion of adjacent structures. After washout of cabozantinib, radical resection, including nephrectomy, was successfully performed without any major complications, either intra-operative or perioperative. Negative margins were achieved.

**Conclusions:**

This is a report of neoadjuvant cabozantinib downsizing a tumor and enabling surgical resection in this patient with locally advanced RCC. Our findings demonstrate that neoadjuvant cabozantinib to facilitate subsequent surgical resection may be a feasible option for patients presenting with unresectable RCC.

## Introduction

Cabozantinib is a potent multikinase agent that inhibits, in addition to VEGF receptors, MET, and AXL, both of which are associated with resistance to VEGF-directed therapy. The METEOR phase 3 clinical trial results proved that treatment with cabozantinib increased overall survival, delayed disease progression, and improved the objective response compared with everolimus in advanced renal cell carcinoma (RCC) patients (1). These promising results led to initial approval of cabozantinib treatment for advanced renal cell carcinoma. In the CABOSUN phase 2 clinical trial, cabozantinib treatment demonstrated a significant clinical benefit in progression free survival and objective response rate over standard-of-care sunitinib as first-line therapy in patients with intermediate- or poor-risk metastatic RCC (2). Thus, recently, cabozantinib has been approved for use in the first- and second-line setting in patients with advanced RCC (3). Therefore, cabozantinib was selected to be administered for this patient with locally advanced RCC.

Targeted therapies, primarily inhibitors of the VEGF receptor tyrosine kinase and rapamycin pathways, have changed the management of advanced RCC. Over the past 10 years, studies have established efficacy and have led to approval of sorafenib, sunitinib, temsirolimus, everolimus, pazopanib, axitinib, and also cabozantinib. These agents have significantly improved progression-free survival, with certain therapies achieving a median overall survival of >2 years in advanced RCC patients (4, 5). Most recently, trials on novel multikinase inhibitors, such as cabozantinib, and PD-1 inhibitors, such as nivolumab, have demonstrated significantly prolonged progression free survival and increases in overall survival, compared to standard therapy in metastatic RCC patients (2, 3). Using these agents in the neoadjuvant setting has emerged as a treatment option for locally advanced RCC patients. Neoadjuvant therapy can potentially downsize advanced tumors, enabling surgical interventions when they may not otherwise have been feasible or safe due to unresectable locoregional disease. We describe here, a patient presenting with initially unresectable locally advanced RCC treated with neoadjuvant cabozantinib, downsizing the tumor and enabling surgical resection.

## Case Description

**Figure 1:**
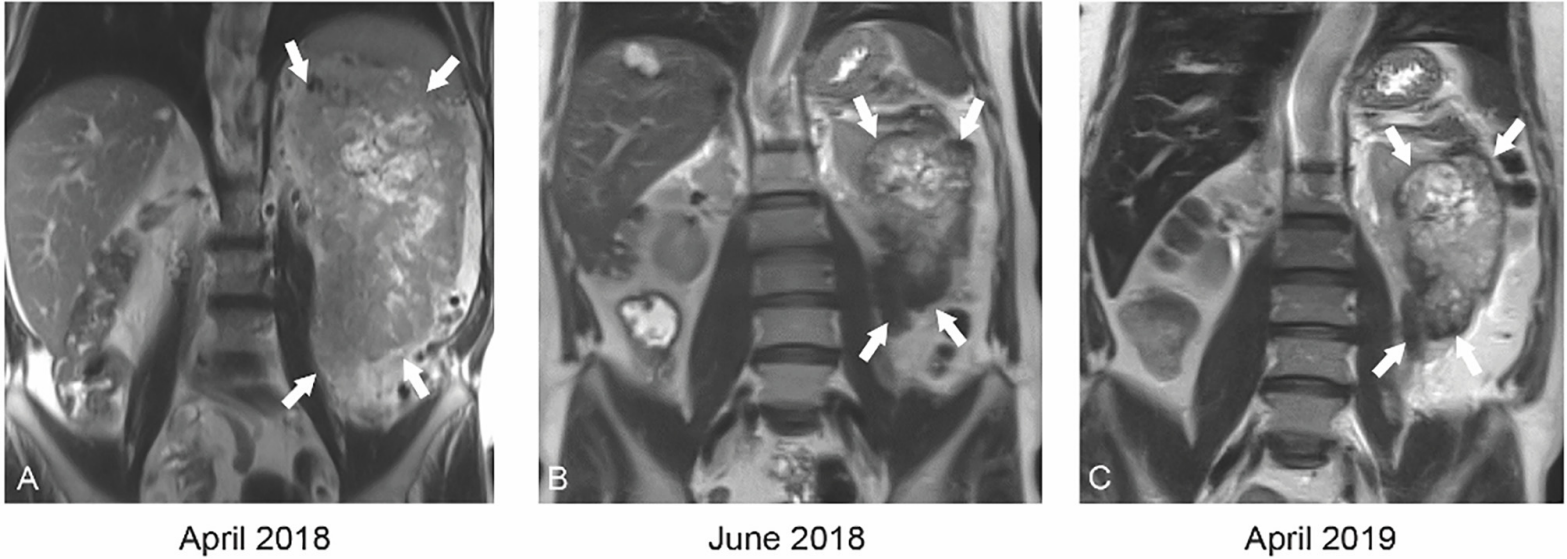
Coronal T2 weighted MRI at baseline **(A)** demonstrates a large 21.7 cm mass (white arrows) replacing the entire left kidney with central areas of necrosis. After just 2 months of cabozantinib therapy **(B)**, the mass had decreased to 12.1 cm (white arrows). After 12 months of cabozantinib **(C)**, the mass was stable in size.

**Figure 2:**
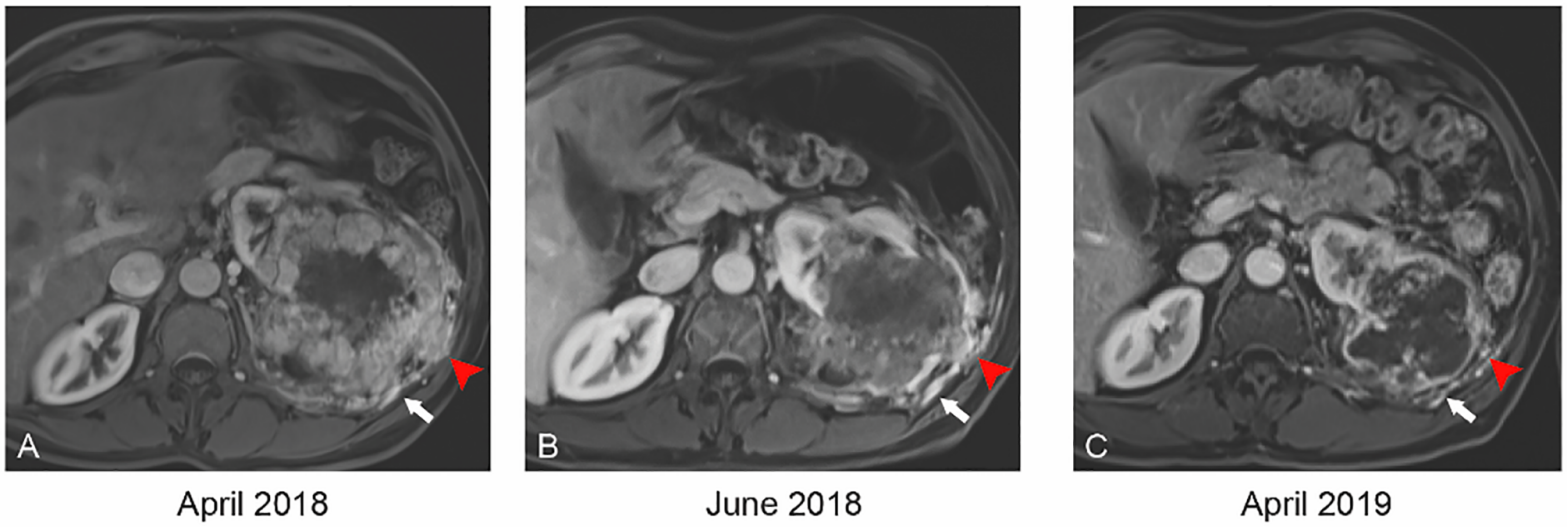
Axial T1 weighted MR with contrast at baseline **(A)** demonstrates tumor invasion into the intercostal space (red arrowhead) and neovascularity (white arrow). By 2 months of therapy **(B)**, the invasion has retracted (red arrowhead). By 12 months **(C)**, the invasion has resolved and vascularity has decreased significantly.

**Figure 3:**
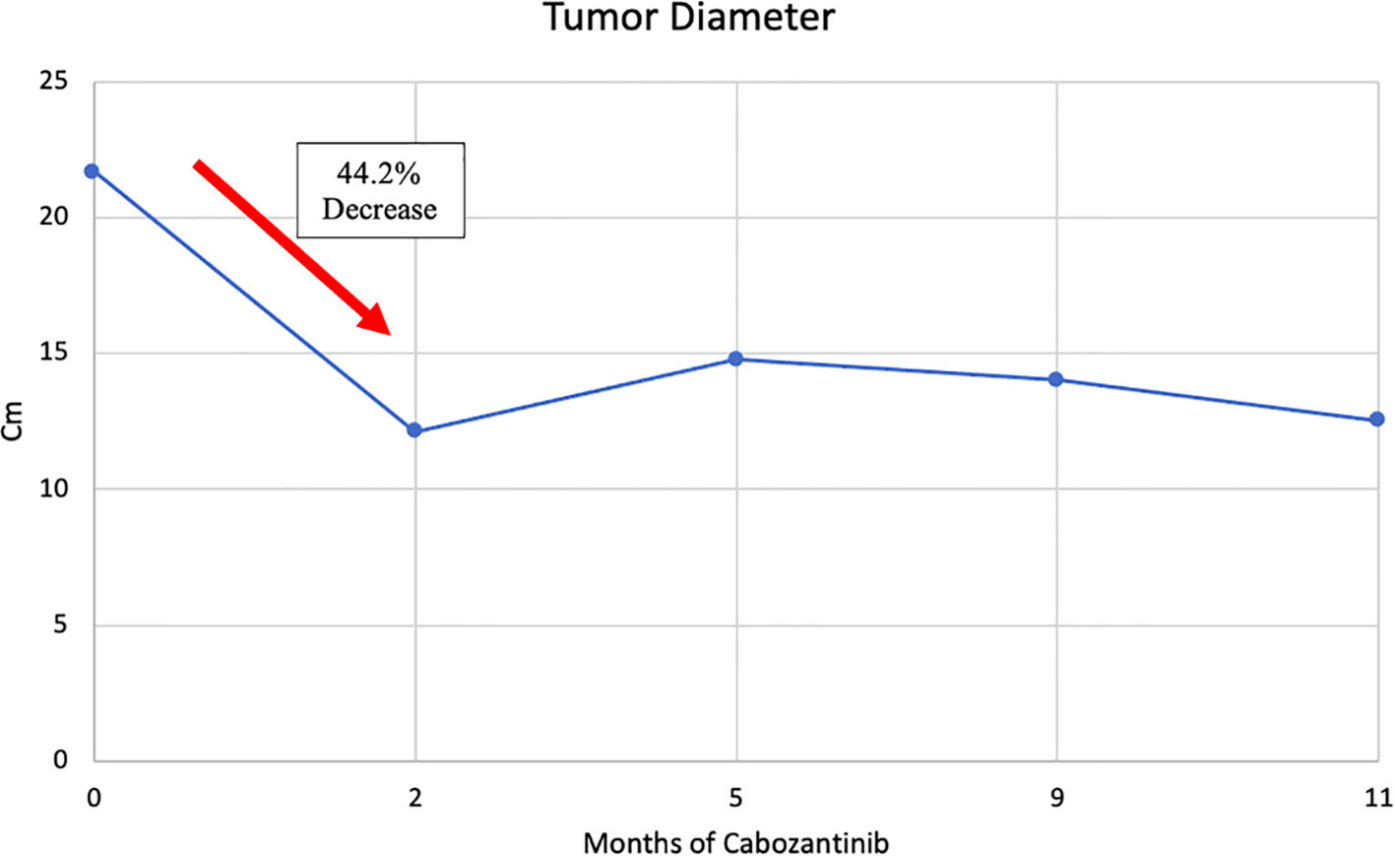
Trend of tumor diameters, from MRIs, during cabozantinib therapy.

A 59-year-old man with an Eastern Cooperative Oncology Group (ECOG) status of 0 presented with a left renal mass in March 2018. Computed tomography (CT) scans of the chest, abdomen, and pelvis revealed a locally invasive 21-cm left renal mass inseparable from the soft tissue of the thorax, psoas muscle, posterior abdominal wall, tail of pancreas, splenic flexure of colon, and inferior margin of spleen with no evidence of nodal involvement or metastatic disease. In April 2018, magnetic resonance imaging (MRI) supported these findings showing a 21.7 cm renal mass invading the renal hilum and adjacent structures described above (**Figure 1A**). In April 2018, patient underwent a CT-guided renal biopsy that confirmed renal cell carcinoma (RCC). The tumor was deemed unresectable at our multidisciplinary genitourinary tumor board, and systemic treatment was recommended. In April 2018, patient was seen in genitourinary medical oncology clinic, and cabozantinib 60 mg daily was started. In June 2018, after 2 months of treatment, MRI revealed a significant decrease in tumor size from 21.7 to 12.1 cm with marked decrease of extension into the psoas muscle, posterior abdominal wall, tail of the pancreas, splenic flexure of the colon, and inferior margin of the spleen (**Figures 1** and **2**). After 11 months of therapy, the corresponding MRI showed grossly stable size of the tumor but resolved invasion of adjacent structures (**Figures 1C**, **2**, and **3**).

During cabozantinib therapy, the patient developed hypertension, secondary to cabozantinib, which was well controlled with lisinopril and amlodipine. Otherwise, there were no adverse events during drug therapy, besides mild hand-foot disease, and patient did not require any dose reduction. The patient was therefore scheduled for surgery after a 3-weeks washout from systemic therapy in March 2019.

**Figure 4:**
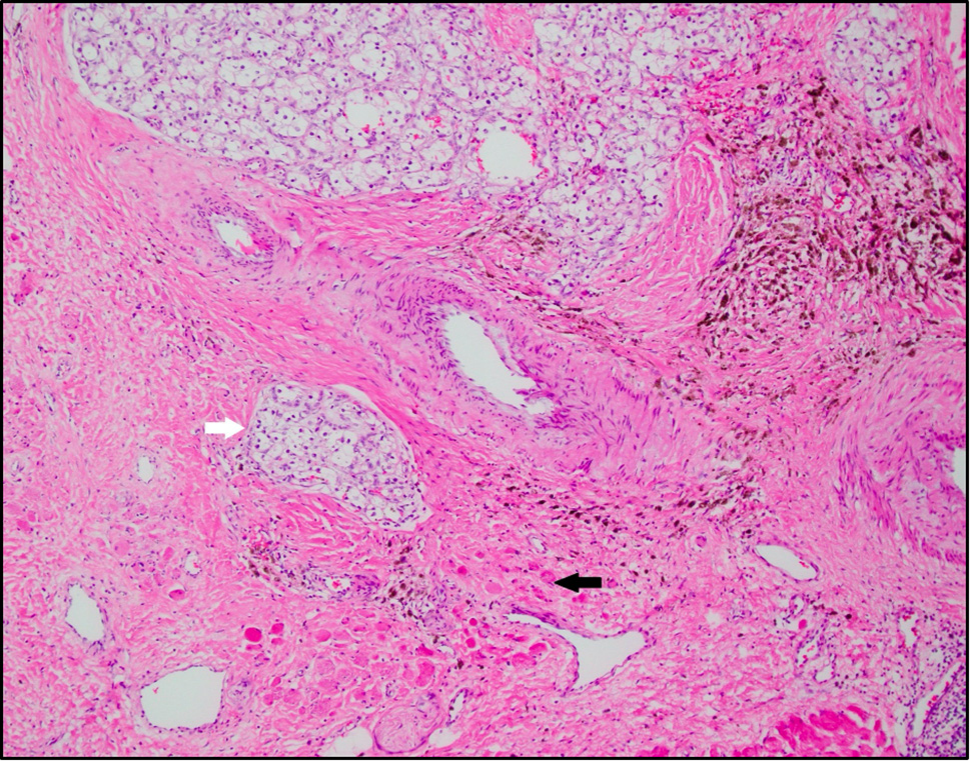
Clear cell renal cell carcinoma (white arrow) infiltrating into skeletal muscle (black arrow), representing psoas muscle and diaphragm. The background is fibrotic with extensive hemosiderin laden macrophages, possibly representing therapy related changes (H&E-10x).

In April 2019, patient underwent *en bloc* left radical nephrectomy, left adrenalectomy, retroperitoneal lymph node dissection, omentoplasty, distal pancreatectomy, splenectomy, and resections of quadratus lumborum, left psoas muscle, left crus muscle, and diaphragm with negative margins. Final pathology confirmed a 13.7 cm T4N0M0 grade 3 clear cell renal cell carcinoma invading the renal vein, renal sinus fat, perinephric fat, and psoas/diaphragm muscle and surgical margins were negative (**Figure 4**). The patient was discharged in a stable clinical status 9 days after surgery. When we wrote this report, the patient was still alive and well, and no evidence of recurrence on imaging.

**Figure 5:**
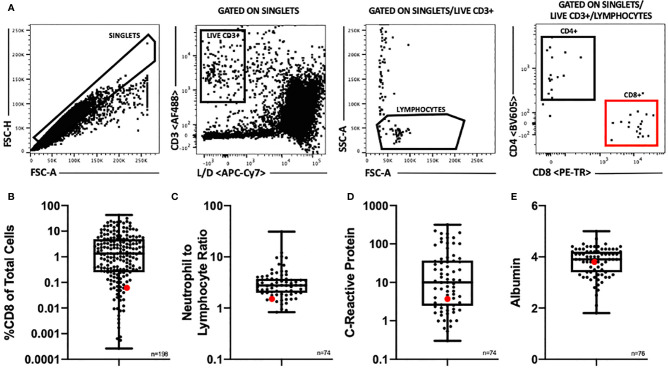
**(A)** Flow cytometry gating scheme. Samples were gated to exclude doublets and cell aggregates and to include only single cells for further analysis. This single cell population was then gated to include only live, CD3+ cells, then to include only lymphocyte sized cells. These gates insured as pure of a T lymphocyte population as possible for further analysis. T lymphocytes were then divided into CD4+ and CD8+ populations. **(B)** Distribution of %CD8 of total cells by flow cytometry among a cohort of renal cell carcinoma patients, n = 198. **(C)** Distribution of pre-operative neutrophil to lymphocyte ratio among a cohort of renal cell carcinoma patients (n=74). **(D)** Distribution of pre-operative C-reactive protein level (mg/L) among a cohort of renal cell carcinoma patients (n=74). **(E)** Distribution of pre-operative albumin level (g/dl) among a cohort of renal cell carcinoma patients (n=76). **(B**–**E)** Patient of interest highlighted in red. Box plots show middle 50% with the median at the center and the whiskers extending to minimum and maximum values.

Correlative studies were performed on resected tumor samples. **Figure 5** shows this patient’s flow cytometry and pre-operative lab results compared to a cohort of renal cell carcinoma patients. The patient’s intraoperative sample was processed to obtain a single cell suspension, which was analyzed using flow cytometry. This allowed for enumeration of tumor infiltrating T lymphocytes (**Figure 5A**). The patient’s tumor had extremely few infiltrating CD8 T cells (0.061% CD8 T cells) (**Figure 5B**), which has been reported to suggest a poor prognosis (6). The patient’s neutrophil to lymphocyte ratio were within the first quartile of the cohort’s results (**Figure 5C**). His pre-operative C-reactive protein level and albumin level were within the second quartile (**Figures 5D, E** **)**.

**Figure 6:**
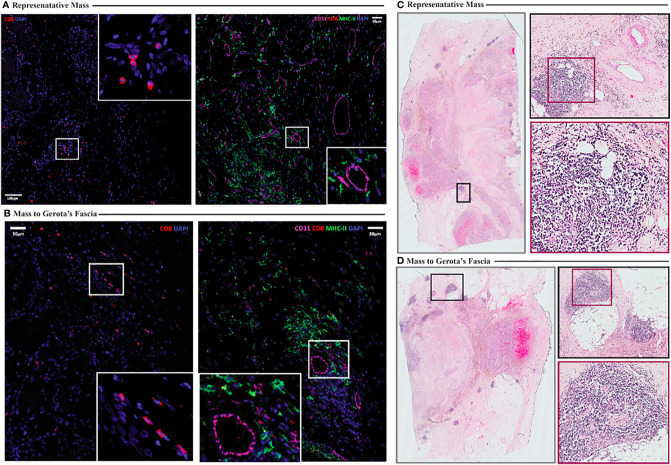
**(A, B)** Immunofluorescence imaging illustrating sparse CD8 T cell infiltration (**A**, **B**, left) and presence of CD31+ endothelium (**A**, **B**, right) in resected tumor specimens (**A** representative. Mass, **B** mass to Gerota’s fascia). **(C, D)** Hematoxylin and eosin staining shows presence of tertiary lymphoid structures (highlighted in insets) (**C** representative mass, **D** mass to Gerota’s fascia).

Correlative studies were also performed on the formaldehyde fixed paraffin embedded pathology specimens from the tumor resection. Immunofluorescence imaging showed sparse CD8 T cell infiltration in two distinct specimens from the resected tumor lesion (**Figures 6A, B** **)**, consistent with flow cytometry results (**Figures 5A, B** **)**. The presence of CD31+ endothelium throughout the tumor specimen was also evident on immunofluorescence imaging (**Figures 6A, B** **)**. Interestingly, tertiary lymphoid structures (TLS) were identified in both specimens examined (**Figures 6C, D** **)**, despite the paucity of CD8 T cells identified on flow cytometry and immunofluorescence imaging, which is consistent with a report that there does not appear to be a correlation between CD8 T cell infiltration and the presence of TLS in RCC tumors (6).

Next generation sequencing testing was performed on intra-operative resected tumor samples. No microsatellite instability was detected. No genes with pathogenic or likely pathogenic alternations were detected in the sample. Of note, there were no mutations detected in MET, VHL, PBRM1, BAP1, SETD2, or RET. Immunohistochemistry results were positive for MLH1 (2+, 80%), MSH2 (2+, 90%), MSH6 (1+, 60%), and PMS2 (1+, 80%) and were negative for PD-L1 (SP142).

## Discussion

We reported a patient presenting with initially unresectable locally advanced RCC treated with neoadjuvant cabozantinib, downsizing the tumor, and enabling surgical resection. This case study demonstrates important points about cabozantinib for patients with advanced RCC.

In patients presenting with initially unresectable advanced RCC, neoadjuvant cabozantinib may be of benefit for downsizing tumors to enable surgical resections that otherwise wouldn’t be possible. We also showed that this approach is feasible and safe prior to surgery. Reduction of tumor size was rapid, as the tumor diameter decreased by 44.2% after only 2 months of cabozantinib, a partial response on the Response Evaluation Criteria for Solid Tumors (RECIST). In the following 9 months, of 11 months total, of cabozantinib therapy, tumor size, appearance, and involvement of surrounding tissues remained stable. It has been suggested that one mechanism of cabozantinib’s efficacy is modulation of the immune microenvironment in the tumor (7–9), so it is interesting that this patient had a strong response to cabozantinib, despite a sparsely immune infiltrated tumor.

Although neoadjuvant therapy shows reduction of tumor size, it is yet not clear whether a prolonged survival can be achieved for patients. Several studies of neoadjuvant therapy for RCC have demonstrated consistent primary tumor size reduction to enable surgical resection (10–13). Rini *et al*. were the first to report response (28% median reduction in primary tumor size) of advanced primary renal tumors to treatment with neoadjuvant sunitinib. Of 28 patients with advanced RCC deemed unsuitable for initial nephrectomy, 13 (45%) were able to undergo nephrectomy following sunitinib (12). Emerging data from recent prospective phase II trials have also reported consistent tumor size reduction facilitating nephrectomies (13–16). In a literature review of neoadjuvant therapy to facilitate nephrectomy for locally advanced disease, Bindayi *et al*. found that 12 of 14 of the studies investigated neoadjuvant sunitinib or sorafenib (17). In a prospective phase 2 clinical trial, Karam *et al*. reported that axitinib, when given prior to surgery, resulted in significant shrinking of kidney cancers, facilitating surgical resections (13). Most recently, Roy et al. reported two patients with unresectable RCCs that were treated with cabozantinib, and achieved >50% tumor shrinkage which allowed surgical resection (18).

In summary, we present a patient with locally advanced RCC, treated with neoadjuvant cabozantinib downsizing a tumor and enabling surgical resection in this patient. Our findings demonstrate that neoadjuvant cabozantinib to facilitate subsequent surgical resection may be a feasible option for patients presenting with unresectable RCC. There is still a need for more effective neoadjuvant agents that might improve outcome of kidney cancer patients. Currently, we are conducting a neoadjuvant cabozantinib clinical trial at our institution (NCT04022343), with multiple correlative studies to facilitate identification of the patients most likely to respond.

## Data Availability Statement

The original contributions presented in the study are included in the article/supplementary materials. Further inquiries can be directed to the corresponding authors.

## Ethics Statement

Written informed consent was obtained from the relevant individual(s), and/or minor(s)’ legal guardian/next of kin, for the publication of any potentially identifiable images or data included in this article.

## Author Contributions

MB and VM conceived and designed the study. JJ, MB, CJ, and VM wrote the manuscript. MB, VM, JB, LH, AS, SM, OK, and BC provided the study materials or patients. LH, HK, CJ, and VM performed the experiments. MB managed the cabozantinib therapy. VM and SM performed the surgery. All authors contributed to the article and approved the submitted version.

## Funding

This paper is supported by grants from the John Robinson Family Foundation, the Christopher Churchill Foundation. CJ is supported by a National Cancer Institute grant (1-F30-CA-243250).

## Conflict of Interest

MB has acted as a paid consultant for and/or as a member of the advisory boards of Exelixis, Bayer, BMS, Eisai, Pfizer, AstraZeneca, Janssen, Calithera Biosciences, Genomic Health, Nektar, and Sanofi and has received grants to his institution from Xencor, Bayer, Bristol-Myers Squibb, Genentech/Roche, Seattle Genetics, Incyte, Nektar, AstraZeneca, Tricon Pharmaceuticals, Genome & Company, AAA, Peloton Therapeutics, and Pfizer for work performed as outside of the current study.

The remaining authors declare that the research was conducted in the absence of any commercial or financial relationships that could be construed as a potential conflict of interest.

## Abbreviations

VEGF, vascular endothelial growth factor; RCC, renal cell carcinoma; CT, computed tomography; MRI, magnetic resonance imaging; TLS, tertiary lymphoid structure.
